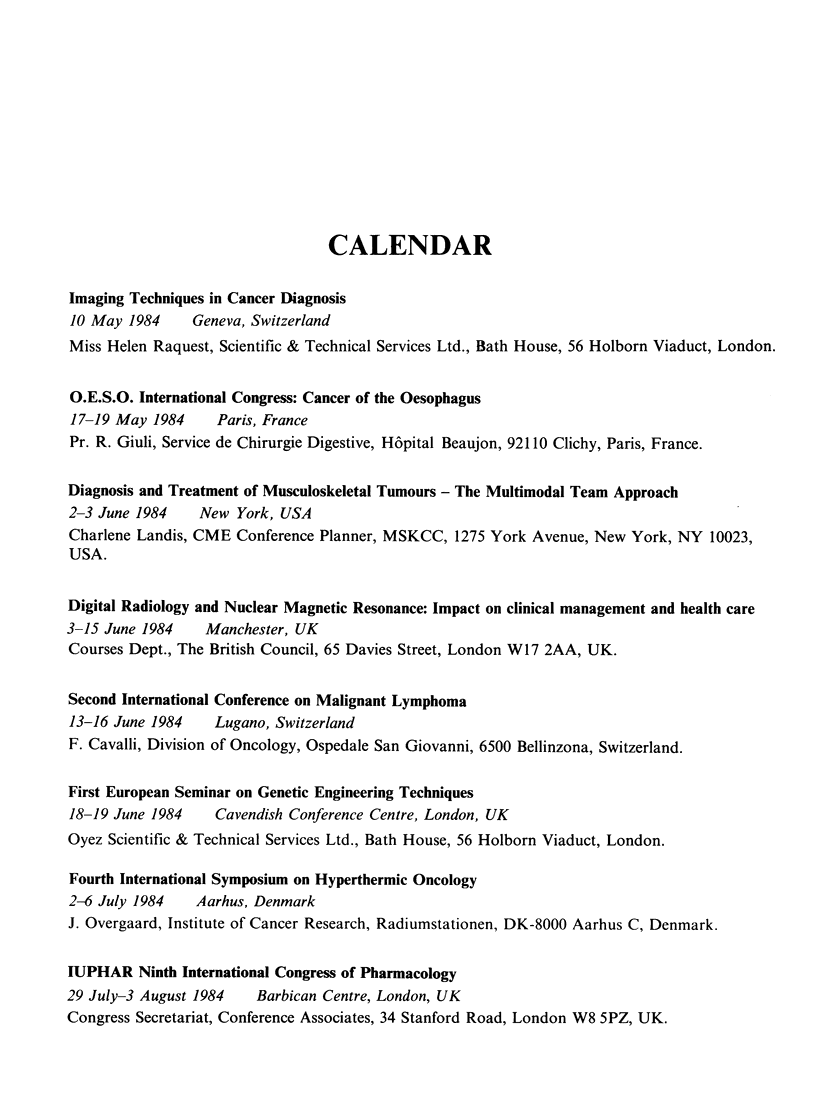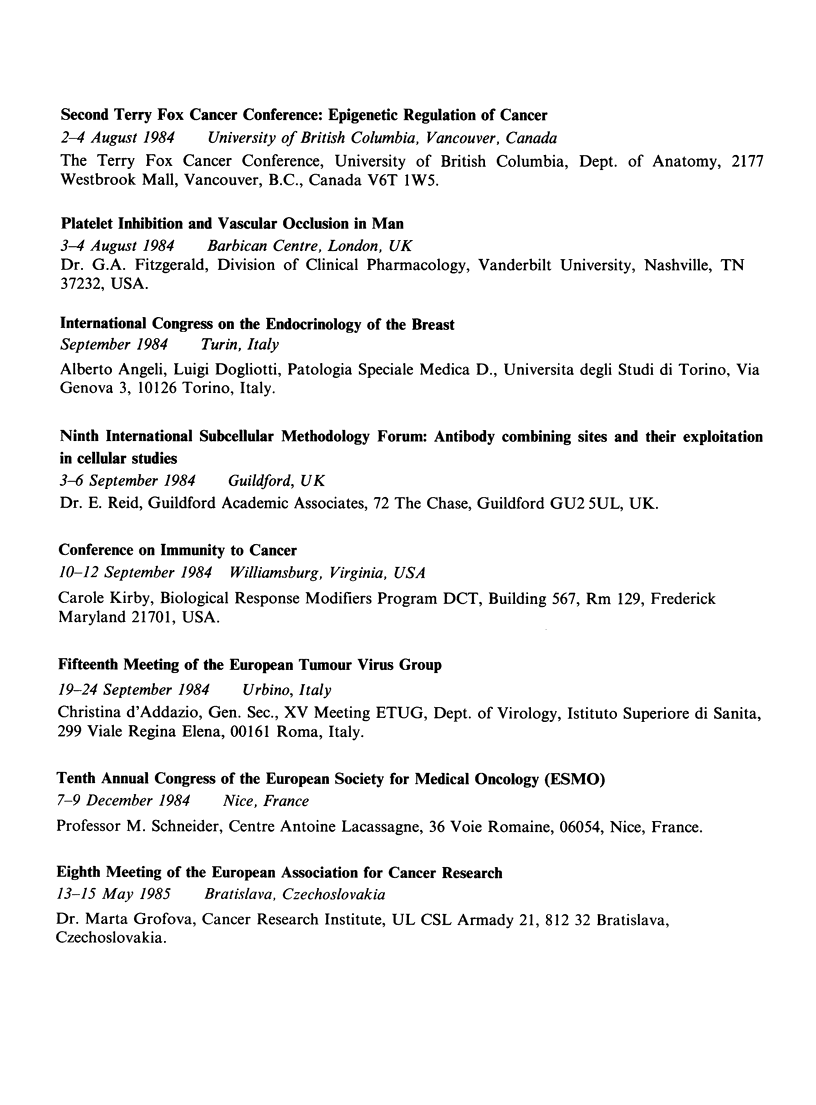# Calendar

**Published:** 1984-04

**Authors:** 


### CALENDAR

**Imaging Techniques in Cancer Diagnosis**
*10 May 1984 Geneva, Switzerland*
Miss Helen Raquest, Scientific & Technical Services Ltd., Bath House, 56 Holborn Viaduct, London.

**O.E.S.O. International Congress: Cancer of the Oesophagus**
*17–19 May 1984 Paris, France*
Pr. R. Giuli, Service de Chirurgie Digestive, Hôpital Beaujon, 92110 Clichy, Paris, France.

**Diagnosis and Treatment of Musculoskeletal Tumours – The Multimodal Team Approach**
*2–3 June 1984 New York, USA*
Charlene Landis, CME Conference Planner, MSKCC, 1275 York Avenue, New York, NY 10023, USA.

**Digital Radiology and Nuclear Magnetic Resonance: Impact on clinical management and health care**
*3–15 June 1984 Manchester, UK*
Courses Dept., The British Council, 65 Davies Street, London W17 2AA, UK.

**Second International Conference on Malignant Lymphoma**
*13–16 June 1984 Lugano, Switzerland*
F. Cavalli, Division of Oncology, Ospedale San Giovanni, 6500 Bellinzona, Switzerland.

**First European Seminar on Genetic Engineering Techniques**
*18–19 June 1984 Cavendish Conference Centre, London, UK*
Oyez Scientific & Technical Services Ltd., Bath House, 56 Holborn Viaduct, London.

**Fourth International Symposium on Hyperthermic Oncology**
*2–6 July 1984 Aarhus, Denmark*
J. Overgaard, Institute of Cancer Research, Radiumstationen, DK-8000 Aarhus C, Denmark.

**IUPHAR Ninth International Congress of Pharmacology**
*29 July–3 August 1984 Barbican Centre, London, UK*
Congress Secretariat, Conference Associates, 34 Stanford Road, London W8 5PZ, UK.

**Second Terry Fox Cancer Conference: Epigenetic Regulation of Cancer**

*2–4 August 1984* *University of British Columbia, Vancouver, Canada*

The Terry Fox Cancer Conference, University of British Columbia, Dept. of Anatomy, 2177 Westbrook Mall, Vancouver, B.C., Canada V6T 1W5.

**Platelet Inhibition and Vascular Occlusion in Man**

*3–4 August 1984* *Barbican Centre, London, UK*

Dr. G.A. Fitzgerald, Division of Clinical Pharmacology, Vanderbilt University, Nashville, TN 37232, USA.

**International Congress on the Endocrinology of the Breast**

*September 1984* *Turin, Italy*

Alberto Angeli, Luigi Dogliotti, Patologia Speciale Medica D., Universita degli Studi di Torino, Via Genova 3, 10126 Torino, Italy.

**Ninth International Subcellular Methodology Forum: Antibody combining sites and their exploitation in cellular studies**

*3–6 September 1984* *Guildford, UK*

Dr. E. Reid, Guildford Academic Associates, 72 The Chase, Guildford GU2 5UL, UK.

**Conference on Immunity to Cancer**

*10–12 September 1984* *Williamsburg, Virginia, USA*

Carole Kirby, Biological Response Modifiers Program DCT, Building 567, Rm 129, Frederick Maryland 21701, USA.

**Fifteenth Meeting of the European Tumour Virus Group**

*19–24 September 1984* *Urbino, Italy*

Christina d'Addazio, Gen. Sec., XV Meeting ETUG, Dept. of Virology, Istituto Superiore di Sanita, 299 Viale Regina Elena, 00161 Roma, Italy.

**Tenth Annual Congress of the European Society for Medical Oncology (ESMO)**

*7–9 December 1984* *Nice, France*

Professor M. Schneider, Centre Antoine Lacassagne, 36 Voie Romaine, 06054, Nice, France.

**Eighth Meeting of the European Association for Cancer Research**

*13–15 May 1985* *Bratislava, Czechoslovakia*

Dr. Marta Grofova, Cancer Research Institute, UL CSL Armady 21, 812 32 Bratislava, Czechoslovakia.